# Biopsychosocial approaches to a patient with vomiting of 10 years' duration – a case of temporal lobe epilepsy

**DOI:** 10.1186/1751-0759-3-2

**Published:** 2009-01-23

**Authors:** Hiromi Mutsuura, Mikihiko Fukunaga, Kenji Kanbara, Takami Yagyu, Kazumi Yamamoto, Kana Kitamura, Ikumi Ban, Yoshihide Nakai

**Affiliations:** 1 Department of Psychosomatic Medicine, Kansai Medical University, Moriguchi-shi, Osaka, Japan; 2 Neyagawa Sanatorium, Neyagawa-shi, Osaka, Japan

## Abstract

**Background:**

Vomiting is commonly encountered in clinical medicine. When organic gastrointestinal, metabolic, and brain diseases are ruled out, many cases are considered to be functional. We experienced an adult patient with epilepsy whose main symptom was vomiting. Biopsychosocial approaches were needed to control the symptoms.

**Case presentation:**

A 26-year-old female with a 10-year history of persistent vomiting was found to have temporal lobe epilepsy (TLE). Throughout this time, during which the vomiting had become part of a vicious cycle, her epilepsy was poorly controlled by medication. Biopsychosocial approaches were employed successfully and the patient subsequently undertook training to become a home-helper, started a job, and was able to leave her parents' house and live independently. All of her symptoms resolved after she became self-sufficient.

**Discussion:**

Vomiting without impaired consciousness is seldom considered to be a manifestation of epilepsy. Difficulty in recording an electroencephalogram (EEG) because of the presence of persistent vomiting delayed the diagnosis. The improvement of symptoms was thought to have been due to the patient's emotional stabilization and physical improvement, which may have stabilized the limbic system.

**Conclusion:**

When an illness persists for many years and conditioning and a vicious cycle occur secondarily, systematic biopsychosocial approaches are needed in addition to general treatment. Also, secondary symptoms make the diagnosis more difficult when efforts at treatment are ineffective.

## Background

There are many medical causes of vomiting. If the patient is a young adult who has not suffered an injury, does not have an abnormality determined by brain CT or biochemical examinations, and has not experienced convulsions or episodes of loss of awareness, most doctors consider vomiting to be related to a gastrointestinal disorder. If all gastrointestinal organic diseases are ruled out, in many cases the condition is considered to be functional.

Ictal vomiting is usually considered to be a rare clinical manifestation during seizures originating from the temporal lobes [1,2]. Vomiting is severe during the seizure, sometimes accompanied by tightness in the throat or feelings of fear [1,3]. Ictal vomiting is often observed in children [4] with an anterior temporal lobe tumor or hippocampal sclerosis [1,5], but there are some case reports of onset in adults at the age of 20 years [5,6]. Shuper and Goldberg-Stern defined the Latin term "ictus emetics" (ictal vomiting) as "vomiting that appears as the sole or major manifestation of a seizure" in a pediatric case report [7]. Therefore, ictal vomiting can occur in the presence of a variety of disorders.

We treated to an adult patient with persistent episodes of vomiting lasting for several days. The patient's vomiting was not accompanied by impaired consciousness, and the diagnosis of epilepsy was only made 10 years after the vomiting was first reported. During that period, a vicious cycle involving the patient's symptoms and family relationships occurred secondarily, and biopsychosocial approaches to treatment were required. The elements involved in this case are relevant to the importance of systematic biopsychosocial therapy and the difficulty in diagnosing the cause of vomiting in some patients.

## Case Presentation

The patient was a 26-year-old female with a 10-year history of vomiting. She had been hospitalized and had required intravenous hydration more than 30 times for that condition. Vomiting usually started with mild left abdominal pain, and did not subside even when the stomach contents had been emptied resulting in "dry heaves". Episodes usually persisted for several days. Throughout the vomiting episodes, her consciousness was clear. Antiemetic, antianxiety, and anticonvulsant drugs were not effective. When she was 25 years old, she first came to our psychosomatic medicine department where she received medical and behavioral treatments for what was diagnosed as cyclic vomiting syndrome (CVS) [8]; however, her symptoms were poorly controlled. When the patient was 26 years old, she was hospitalized and underwent a thorough medical examination.

### Diagnosis of epilepsy

**Figure 1:**
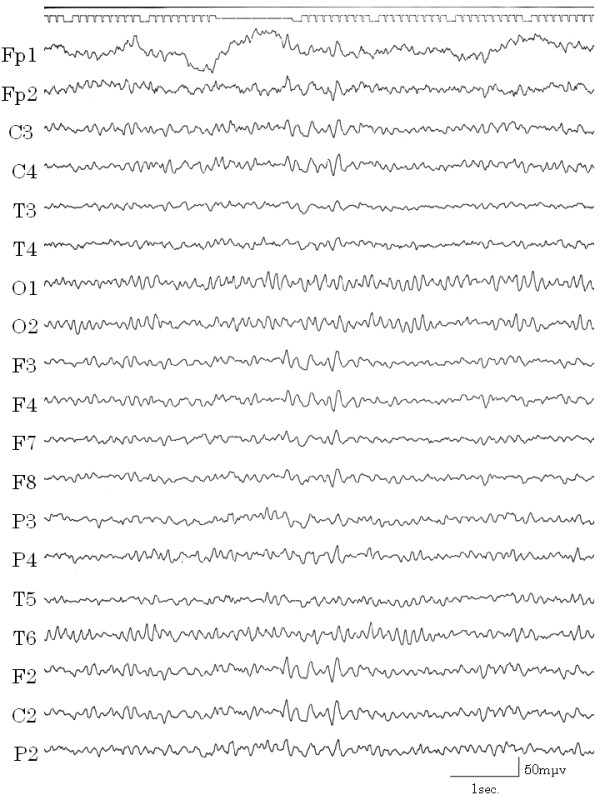
**High amplitude slow waves**. EEG showed high amplitude, 5–6 Hz and slow waves during the vomiting-free periods.

The patient's height was 165 cm and body weight was 55 kg. Her past and family medical history was noncontributory. The results of physical and neurological examinations, esophageal-gastrointestinal endoscopy, and routine biochemical examinations were all normal. She had not experienced external injuries, convulsion, loss of awareness, or migraine headaches. Electroencephalograms (EEGs) were recorded from 19 electrodes (International 10/20 System) with A1+A2 as the recording reference. The patient kept both eyes closed during the recording. The recording was done approximately 500 times. On EEGs performed at high amplitude, 5~6 Hz waves and slow waves (Figure 1) were evident but there were no sudden abnormal waves. In the background, 9–10 Hz alpha waves were recorded around the occipital lobes. Results of brain emission computed tomography (CT) and single photon emission CT (SPECT) of the brain were normal and no atrophy or tumors were evident.

**Figure 2:**
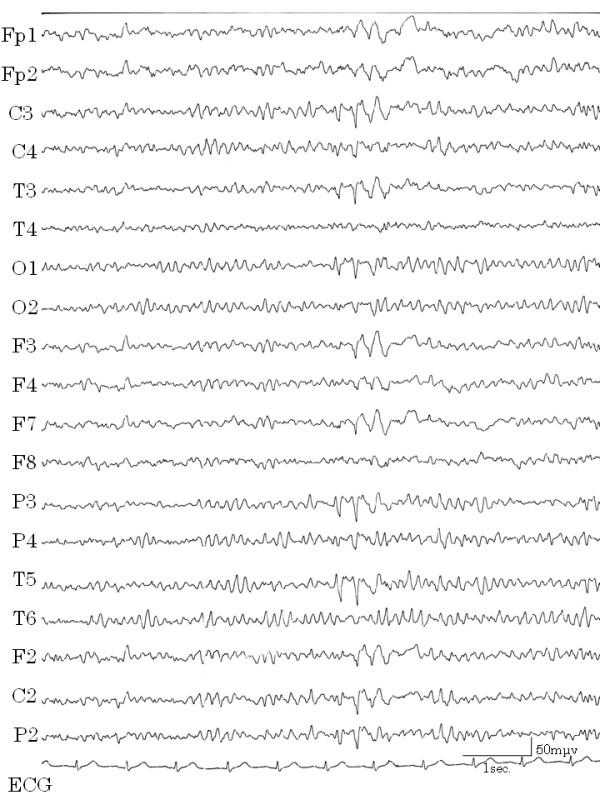
**High amplitude slow waves correlated with vomiting**. EEG with light symptoms. The increase in irregular waves correlated with vomiting. EEGs during severe vomiting could not be recorded properly.

**Figure 3:**
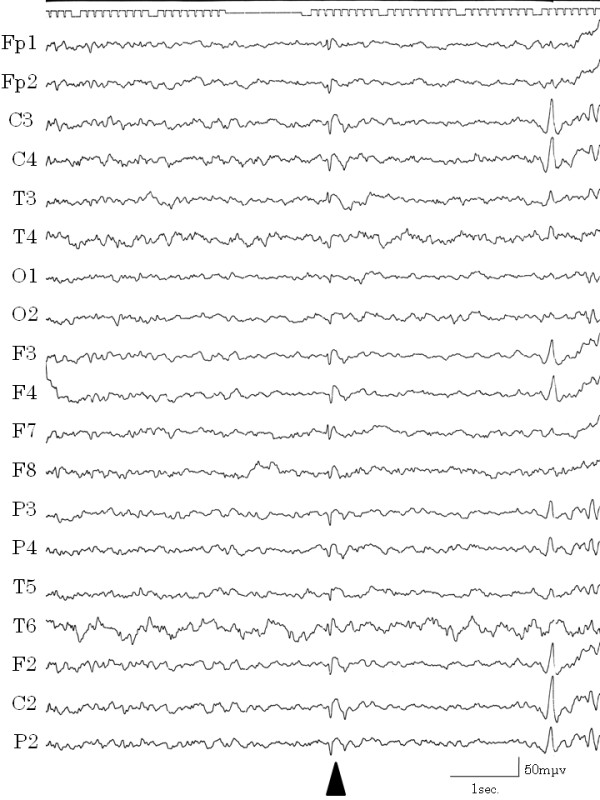
**Spike and wave complexes**. Spike and wave complexes were recorded in T3 and F7 (temporal lobe) when the patient was drowsy.

**Figure 4:**
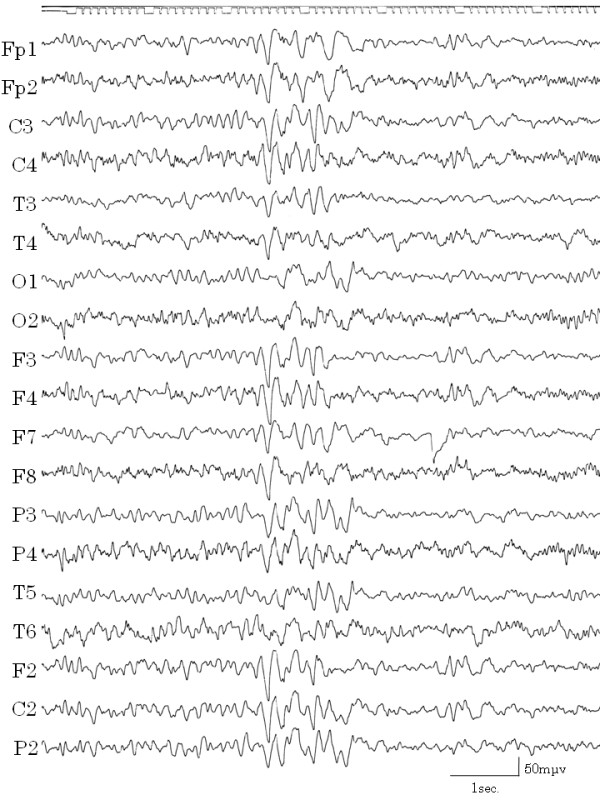
**Irregular slow wave burst**. Irregular slow wave bursts were recorded repeatedly when the patient was drowsy.

**Figure 5:**
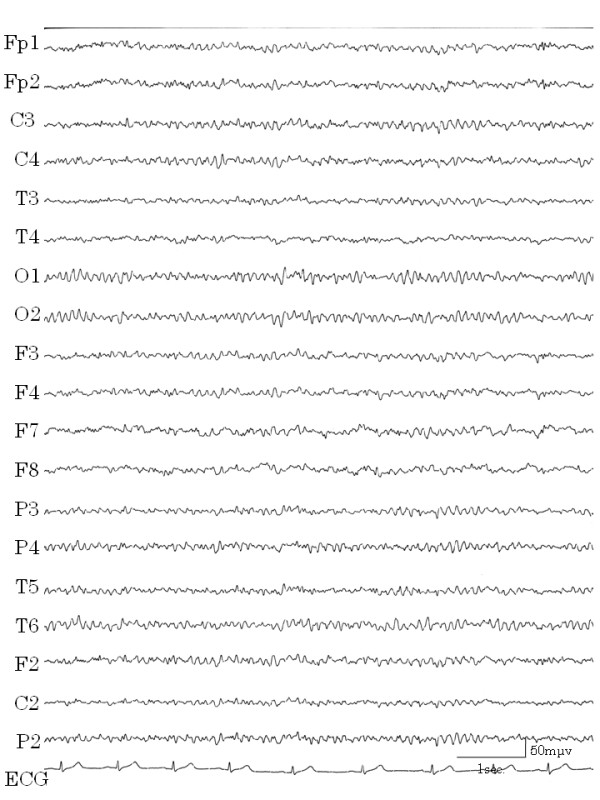
**EEG with administration of clonazepam**. Slow waves decreased and changed to regular background waves four months after administration of clonazepam 1 mg.

During one hospital stay, the patient complained that she could not speak smoothly, had cramping of the tongue, and clenched her teeth against her will. These symptoms subsided naturally within a few hours. After this episode, we learned that she had experienced such cramping twice previously. We considered this to be a symptom of automatism. Although consciousness was not impaired and haloperidol was not effective in stopping the vomiting, epilepsy was cited as a possible diagnosis. After recording EEGs several times, a high amplitude slow wave was obtained while the patient was awake, which correlated with the vomiting (Figure 2). When she was drowsy, spike and wave complexes appeared in the left temporal lobe (Figure 3) and repeated irregular slow wave bursts (Figure 4) were recorded. Through the administration of from 1 to 2 mg clonazepam, background wave activity improved substantially (Figure 5) and vomiting episodes decreased, although the spike and wave complex was noted with drowsiness.

### Biopsychosocial approaches

On first consulting with this patient, we suspected that her illness had a complex etiology and that both psychological and medical approaches to treatment would be required. We reasoned that a psychological problem might be a trigger for her episodes of epilepsy. The patient's family and previous doctors had told her that her illness was caused by mental weakness. She had not attended college nor had she ever been employed, and as a result she had no hope for her future. She was in despair over her health, gave up being understood by others, and would not confide in others. She unconsciously needed the symptoms to escape from reality. We used both physical and psychological approaches in her treatment, involving relaxation techniques, breathing exercises, drawing pictures and counseling. She slowly started to discuss the family situation that had begun in childhood and had continued to the present. She said that her mother often disagreed with what she wanted to do and she began to think that it was no use thinking for herself. Her life at that time was filled with a sense of "abandonment". After she made a friend in the hospital and opened her heart to others, she began to speak tearfully in the therapeutic sessions. She said that she was aware that her symptoms had not been due to a physical problem but rather a mental one, and she expressed the feeling that she would be better off living away from her family. As a child she had been well behaved, and her mother paid little attention to her because she was busy caring for her elder sister, who easily got colds and became tired. When her elder sister entered college and was seldom at home, the mother began nagging the patient to do certain things and was overprotective of her. After several therapeutic sessions, her attitude was more positive. To support her "to do things on her own", we intervened with the family, who were of the opinion that "the patient with the illness was wrong". We tried to encourage the patient to become independent and to separate from the family, and we asked the father and sister to assist in that effort. We complimented the patient's mother for her efforts in caring for her daughter. When she left the hospital, she no longer felt "abandonment" but instead was committed to "live her own life by herself".

**Figure 6:**
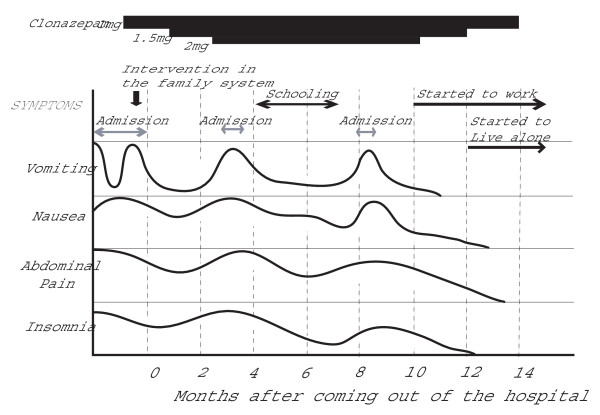
**Changes in symptoms**. Nausea decreased when the patient began to attend school for job training, although her symptoms worsened with fatigue. Insomnia and vomiting had resolved when she started her job. All symptoms had disappeared when she began to live alone and support herself.

**Figure 7:**
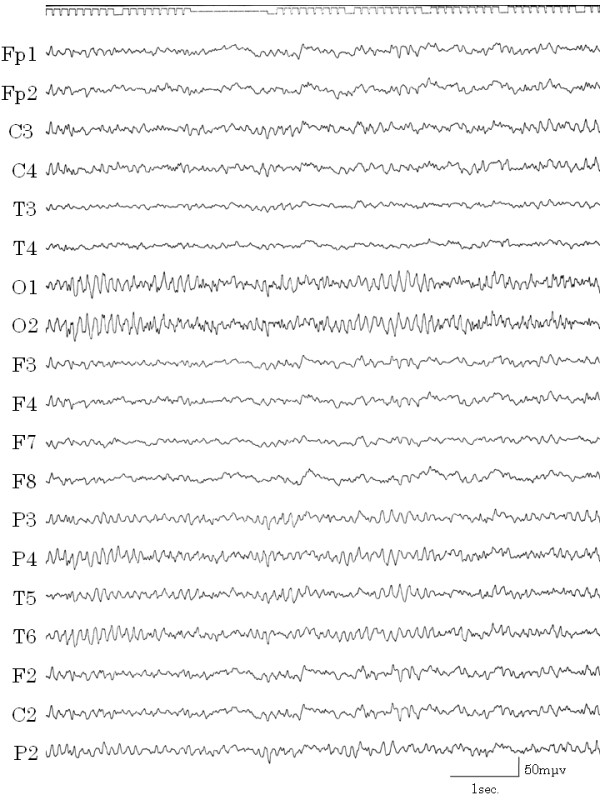
**EEG after treatment**. EEG after implementing biopsychosocial approaches. The patient had no vomiting and had discontinued medication. Irregular waves were evident in the background, although there were no high amplitude slow waves.

After she left the hospital, she had some ictal vomiting when excessively stressed. We listened to her with empathy and acceptance, supported her both mentally and physically, and assisted her in establishing independence. Her symptoms markedly improved when she obtained job training as a home-helper and subsequently found employment. When she began to live alone and support herself, all of her symptoms disappeared (Figure 6). Within several months she was able to decrease and finally discontinue medication and no longer required medical care. An EEG while the patient was awake and unmedicated showed irregular waves in the background and there were no high amplitude slow waves (Figure 7). She has been in good health without requiring medication for more than seven years.

## Discussion

We reasoned that the patient, who had had epileptogenesis from an early age, fell ill when her mother began to interfere with her life and overprotect her. The stress resulting from this situation was the likely trigger and modulator of her epileptic seizures. Because of her illness, she had little connection with society and her relationship with her mother had deteriorated greatly. As a child, she was considered to be "a good girl" and her mother gave more attention to her elder sister. When she fell ill, she might have had ambivalent feelings of joy over the mother's concern and rebellion against her mother's interference, and she may have then somaticized her ambivalence. As her mother often ignored her since childhood, she had fallen into an escape behavior pattern and retreated into illness when faced with stressors. As a behavioral pattern for avoiding problems, she was gradually conditioned to exhibit vomiting when she was overly stressed, and she then developed repeated episodes of vomiting. In addition, the uncontrolled vomiting from her epilepsy that occurred in the absence of stress increased her feelings of helplessness. If the vomiting were solely a psychological symptom, she might have more easily controlled and understood it. Such feelings of lack of control became an additional trigger for her epilepsy: A vicious cycle was formed. When the vomiting began, the epilepsy may have been at the core. However, after about 10 years, the vomiting had become part of a vicious cycle, and her epilepsy was poorly controlled by medication alone. Integrated treatments that involved nurturing her immature personality mentally and socially and supporting the growth of all family members who were in the vicious cycle were needed. After the diagnosis of TLE and use of medication, the patient's EEG results improved but vomiting still occurred in the presence of stress. All symptoms resolved when she became independent through our support, though the spike and wave complexes remained with drowsiness.

The improvement of symptoms was thought to be due to the patient's emotional stabilization and physical improvement, involving sleep, nutrition and physical activity, which may have stabilized the limbic system. The end result was that the epileptic threshold was raised and it was possible to control the patient's symptoms without medication.

Since components of the limbic system mediate emotional experiences, and memory and behavioral responses, patients with epileptic disturbances in these structures might be particularly vulnerable to the triggering of seizures by certain stimuli that evoke emotional responses [9]. Recently, Eggers proposed a circuit of stress (or circuit of emotion), which runs from the hippocampus to the amygdala, dorsal raphe nucleus, entorthinal cortex, and then back to the hippocampus [10]. Eggers also noted that the central pathophysiology of both chronic stress and TLE (the most common cause of intractable adult epilepsy) is cell loss in the dentate, and psychological stress is a frequent trigger for TLE [11]. Intense emotional reactions [12], "tension and anxiety", and "unhappiness/depression" [13] are reported to be seizure inducing factors, while "relaxation" and "happiness" are associated with decreased seizure frequency [13]. Williams et al. reported that approximately 70% of patients whose seizures appeared to be precipitated at times by emotional stress and were not controlled by anticonvulsant medication demonstrated substantial improvement in seizure control after psychiatric treatment, while 32% had their anticonvulsant medication dose reduced and 16% discontinued medication altogether [14]. Psychological therapies are helpful for the management of poorly controlled epilepsy [15,16]. A relationship between EEG and stress was reported in a 1959 study which found that psychological stress was associated with EEG abnormalities [17]. All of the aforementioned studies are consistent with our hypothesis in this case report.

This case was not typical TLE, because the main symptom was persistent vomiting, and the vomiting continued for several days. This made the diagnosis more difficult. Another difficulty in this patient was performing an EEG in the presence of severe vomiting, which also delayed the diagnosis.

Gastrointestinal symptoms that continue for several hours have been noted previously in patients with TLE. Yukselen [18] reported a case of psychomotor epilepsy, with a partial seizure, after a traffic accident in an adult whose symptoms were abdominal pain lasting for several hours followed by nausea and vomiting. Peppercorn [19] reported a case of abdominal epilepsy with temporal region EEG abnormality, in which the symptoms were nausea sustained for several hours and severe dizziness. Cases of abdominal pain in patients with epilepsy (abdominal epilepsy; a form of TLE) have also been reported [19-21]. To the best of our knowledge, there has not been an adult case report of persistent vomiting that continued for several days without alteration of consciousness. There have been reports of two pediatric cases with symptoms in common with our case [22,7]. The case that most closely resembled ours was a child with migraine headaches and paroxysmal vascular abnormality in the temporal regions. Repeated vomiting episodes lasting for 2 to 10 days occurred without seizures. Ictal EEGs showed delta activity in the front-temporal areas, and SPECT showed decreased tracer uptake in both temporal regions during the attack-free period [22]. Another pediatric case was a child with epilepsy who had recurrent episodes of severe vomiting lasting for a day and sometimes associated with unusual behavior suggestive of fright. EEG during presentation of symptoms showed slow and sharp waves origination from the left front-temporal regions [7]. Our case and both of these pediatric cases had common foci of abnormal EEG in the temporal lobes.

This case would have fulfilled the criteria of CVS if the diagnosis of epilepsy had not been made. Although the etiology is not clearly known, there may be specific subgroups of CVS that have different etiologies, such as migraine, metabolic, neuroendocrine, and gastrointestinal mechanisms [23]. In other reports of adult CVS cases, most patients did not have an EEG examination [24]. Therefore, it is possible that EEG abnormalities may be present among CVS cases.

## Conclusion

We reported a patient with TLE whose main symptom was persistent vomiting. This case suggests that there may be other illnesses of unknown etiology that are associated with EEG abnormalities. Also, when an illness is persistent, conditioning and a vicious cycle occur as secondary phenomena. At this stage, systematic biopsychosocial approaches are needed in addition to general treatment of the core disease. Also, the conditioning and vicious cycle that occur secondary to the primary disease make the diagnosis more difficult.

## Consent

Written informed consent was obtained from the patient for publication of this case report and the accompanying images. A copy of the written consent is available for review from the Editor-in-Chief of this journal.

## Authors' contributions

Hiromi Mutsuura carried out the medical treatment and drafted the manuscript. Mikihiko Fukunaga, Kenji Kanbara and Yoshihide Nakai helped to draft portions of the manuscript pertaining to biopsychosocial approaches. Takami Yagyu made the diagnosis of this case, carried out the EEG interpretation and assisted with portions of the manuscript concerning epilepsy. Kazumi Yamamoto, Kana Kitamura and Ikumi Ban provided advice for the preparation of this case and English language assistance.

All authors read and approved the final manuscript.
